# Veterinary-specific breakpoints for ceftiofur applicable to canine *Escherichia coli*

**DOI:** 10.1128/jcm.00299-26

**Published:** 2026-07-27

**Authors:** Xukun Dang, Siyu Chen, Hening Lyu, Zhiyu Zou, Da Sun, Lin Yan, Tongran Wang, Chuangpeng Huang, Yifei Li, Shizhen Ma, Rina Bai, Junyao Jiang, Weishuai Zhai, Lu Wang, Fupin Hu, Congming Wu, Mark G. Papich, Jianzhong Shen, Marilyn N. Martinez, Stefan Schwarz, Zhaofei Xia, Yang Wang

**Affiliations:** 1 Technology Innovation Center for Food Safety Surveillance and Detection (Hainan), Sanya Institute of China Agricultural University 833814, Sanya, China; 2 State Key Laboratory of Veterinary Public Health Safety, College of Veterinary Medicine, China Agricultural University 34752, Beijing, China; 3 Department of Clinical Veterinary Medicine, College of Veterinary Medicine, China Agricultural University 34752, Beijing, China; 4 Veterinary Teaching Hospital, China Agricultural University 34752, Beijing, China; 5 Key laboratory of Animal Antimicrobial Resistance Surveillance, Ministry of Agriculture and Rural Affairs, College of Veterinary Medicine, China Agricultural University 34752, Beijing, China; 6 State Key Laboratory for Zoonotic Diseases, Key Laboratory for Zoonosis Research of the Ministry of Education, College of Veterinary Medicine, Jilin University 623721, Changchun, China; 7 Department of Preventive Veterinary Medicine, College of Veterinary Medicine, China Agricultural University 34752, Beijing, China; 8 Institute of Antibiotics, Huashan Hospital, Fudan University 12478, Shanghai, China; 9 College of Veterinary Medicine, North Carolina State University 6798, Raleigh, North Carolina, USA; 10 Center for Veterinary Medicine, Office of Generic Animal Drug, US Food and Drug Administration 824436, Rockville, Maryland, USA; 11 Institute of Microbiology and Epizootics, Centre for Infection Medicine, School of Veterinary Medicine, Freie Universität Berlin 9166, Berlin, Germany; 12 Veterinary Centre for Resistance Research (TZR), School of Veterinary Medicine, Freie Universität Berlin 9166, Berlin, Germany; University of California Davis, Davis, California, USA

**Keywords:** ceftiofur, breakpoint, canine, *Escherichia coli*, PK/PD

## Abstract

Ceftiofur is a third-generation cephalosporin approved for veterinary applications and is used to treat *Escherichia coli* infections in dogs, particularly urinary tract infections (UTIs), in countries including China. Despite this use, there are no approved breakpoints for interpretation of ceftiofur susceptibility testing results. This absence limits the guidance that veterinarians need to effectively prescribe ceftiofur in dogs. It also limits the analysis of data from resistance monitoring and surveillance programs. This study aimed to establish ceftiofur interpretive categories and breakpoints that can be used to assess antimicrobial susceptibility testing results for canine *E. coli* isolates. Our methods included a definition of the wild-type cutoff (CO_WT_) based on resistance phenotypes from two sources, deriving the pharmacokinetics/pharmacodynamics (PK-PD) cutoff (CO_PD_) through PK-PD analyses and Monte Carlo simulations and determining the clinical cutoff (CO_CL_) from a trial in dogs. Minimal inhibitory concentration (MIC) data were collected from China and the European database (European Committee on Antimicrobial Susceptibility Testing [EUCAST]). CO_WT_ was determined as 1 μg/mL. The CO_PD_ was 0.25 μg/mL. Through a canine cystitis trial, a CO_CL_ of 2 μg/mL was determined. Based on these findings and Clinical and Laboratory Standards Institute (CLSI)-recommended methods, we propose urinary tract infection-specific MIC breakpoints of ≤1 µg/mL (susceptible), 2 μg/mL (intermediate), and ≥4 µg/mL (resistant) and corresponding disk diffusion zone diameter breakpoints, determined by the error rate-bounded (ERB) method, of ≥23 mm (susceptible), 20–22 mm (intermediate), and ≤19 mm (resistant). The systemic breakpoints (non-urine) were determined to be ≤0.125 µg/mL (susceptible), 0.25 μg/mL (intermediate), and ≥0.5 µg/mL (resistant).

**IMPORTANCE:**

Ceftiofur can be legally used, but laboratories and veterinarians lack canine-specific breakpoints to determine whether an isolate is susceptible or resistant. Without such criteria, test results can be inconsistent, treatment choices uncertain, and early signs of emerging resistance overlooked. This study fills that gap by defining evidence-based, canine-specific breakpoints for ceftiofur, particularly for urinary tract infections (UTIs). Adoption of these breakpoints will allow more consistent reporting by diagnostic laboratories, improve therapeutic decision-making for veterinarians, and provide a reliable basis for resistance surveillance. These advances strengthen the One-Health antimicrobial stewardship and help preserve the effectiveness of important antimicrobials for companion animals and the wider community.

## INTRODUCTION

With the growing pet population, antimicrobial use in companion animals has attracted growing attention (1). Close human–pet contact raises concerns about cross-species transmission of resistant bacteria, underscoring the need for a One-Health approach (2). *Escherichia coli* is the most common urinary tract pathogen in dogs, accounting for one-third to over half of canine urinary tract infection (UTI) isolates (3), and may also be associated with reproductive tract, respiratory tract, and skin/soft-tissue infections (4). Resistance to first-choice agents, such as amoxicillin, amoxicillin-clavulanate, tetracyclines, first-generation cephalosporins, and trimethoprim-sulfamethoxazole, may limit treatment options in some dogs (5, 6) and increase the use of antimicrobials not approved for veterinary applications. In China, both subcutaneous (SC) and intravenous (IV) formulations of ceftiofur sodium are clinically available and commonly used in veterinary practice. Ceftiofur is widely used in China for its activity against gram-negative bacteria of the order Enterobacterales. In both China and the United States, ceftiofur has been approved for treating canine lower urinary tract infections caused by *E. coli* or *Proteus mirabilis* at a dose of 2.2 mg/kg SC once daily (7). Following ceftiofur sodium SC injection at a concentration of 2.2 mg/kg, model-based simulations predict that total ceftiofur equivalents (protein-bound and -unbound) are ≥0.5 µg/mL for ~30 h in dogs (8) and that mean urine concentrations will exceed 1 µg/mL for 24 h (9).

Antimicrobial susceptibility testing uses interpretive categories and associated breakpoints to classify bacterial isolates as susceptible (S), susceptible dose-dependent (SDD), intermediate (I), non-susceptible (NS), or resistant (R), thereby guiding therapy (10, 11). Optimally, breakpoints should be established before an antimicrobial is introduced into clinical use and should be periodically updated when new pharmacokinetic (PK) and dosing protocols emerge (12). The Clinical and Laboratory Standards Institute (CLSI; https://clsi.org/) and the European Committee on Antimicrobial Susceptibility Testing (EUCAST; https://www.eucast.org/) derive robust interpretive criteria from the combination of *in vitro* studies, pharmacokinetic-pharmacodynamic (PK-PD) modeling, and clinical efficacy data (10). This approach ensures that breakpoints for the category “S” correlate with likely therapeutic success in the target host species (13).

While canine *E. coli* breakpoints are available for several antimicrobials in the 7th edition of CLSI VET01S, they do not exist for ceftiofur (11). Ceftiofur is a third-generation cephalosporin labeled mainly for food animals but approved for, or used as extralabel, in dogs in some countries, including China. The absence of canine-specific CLSI-approved ceftiofur breakpoints hampers the interpretation of ceftiofur minimal inhibitory concentrations (MICs) and limits its use when making therapeutic clinical decisions. Without breakpoints, diagnostic laboratories cannot properly interpret ceftiofur MICs for canine isolates and will not test it on their diagnostic panels.

To address this gap and provide laboratories with the necessary guidance, we established ceftiofur breakpoints for canine *E. coli*. Following current standards (14), we (i) analyzed wild-type MIC distributions to define the wild-type cutoff (CO_WT_), (ii) assessed canine PK-PD cutoff (CO_PD_) to estimate the highest treatable MIC for a given dosing regimen, and (iii) evaluated the MIC-associated clinical outcomes (CO_CL_). We hypothesized that when established, these breakpoints could improve susceptibility classification and support prudent antimicrobial use in canine infections.

## MATERIALS AND METHODS

### Bacterial isolates and identification

Routine clinical samples from infected dogs—including those from the urinary tract, skin, respiratory tract, genital tract, pleural fluid, ascites, hepatobiliary system, and other sites—were collected at the microbiology laboratory of the Veterinary Teaching Hospital at China Agricultural University, Beijing, China, between 2018 and 2023. A total of 880 non-duplicate *E. coli* isolates were recovered using brain heart infusion (BHI) agar supplemented with 5% defibrinated sheep blood. Species identification was confirmed by matrix-assisted laser desorption/ionization time-of-flight mass spectrometry (MALDI-TOF MS; Bruker, USA) and 16S rRNA gene sequencing (15).

The MIC distributions of the isolates from China were compared with publicly available MIC distribution data from the EUCAST website (http://www.eucast.org).

### *In vitro* susceptibility testing: MIC and CO_WT_ determination

The MICs of ceftiofur for the 880 *E. coli* isolates collected in China were determined by the broth microdilution method using two-fold serial dilutions ranging from ≤0.125 to ＞16 µg/mL. Antimicrobial susceptibility testing was performed under standardized laboratory conditions at the Technology Innovation Center for Food Safety Surveillance and Detection (Hainan), Sanya Institute of China Agricultural University, Sanya, China. Commercial ready-to-use microtiter plates manufactured by Thermo Fisher Scientific (Plate code: CHNCARBF) were used in parallel for verification. Inhibition zone diameters around 30 µg ceftiofur disks were additionally measured for a randomly selected subset of 398 isolates from the 880 canine isolates following the recommendations from CLSI VET01S (11). This subset was selected to provide representative coverage of the MIC distribution for MIC-zone diameter correlation and error rate-bounded (ERB) analysis, particularly in the range surrounding the proposed breakpoints, as recommended in CLSI VET02 (16). *E. coli* ATCC 25922 served as the quality control (QC) strain and was tested for its ceftiofur MIC on each self-designed 96-well plate and also on commercial test plates on a daily basis.

The epidemiological cutoff value (ECOFF; also known as the wild-type cutoff, CO_WT_) was calculated using the “ECOFFinder,” which is available as a public access tool (https://clsi.org/meetings/susceptibility-testing-subcommittees/ecoffinder/). The ECOFFinder assumes a log-normal distribution when fitting the MIC data. This fitting procedure is used to identify the wild-type distributions and reports the output for various percentiles (17, 18). This modeling procedure was conducted separately on the isolates from China and EUCAST. Following EUCAST Standard Operating Procedure (SOP) 10.1 guidance, the 97.5% and 99% statistical ECOFFs were reviewed, and a visual inspection of the aggregated histogram was performed to confirm that the putative wild-type mode was not split and that tail proportions were acceptable. When the statistical and visual estimates agreed (or differed by no more than one two-fold dilution), we selected a conservative consensus ECOFF and designated this value as the CO_WT_ for ceftiofur against canine *E. coli*.

### Plasma protein binding assay (canine plasma)

Plasma protein binding of ceftiofur and ceftiofur equivalent metabolites was determined *in vitro* using a microcentrifugation ultrafiltration system. Briefly, pooled blank canine plasma was prepared and aliquoted. Plasma samples were spiked to achieve three nominal concentrations representing low, medium, and high levels (1, 8, and 15 µg/mL, respectively). Spiked plasma replicates were incubated for 30 min at controlled temperature to allow equilibration. After equilibration, 2.0 mL of each spiked plasma sample was transferred into Amicon Ultra-15 centrifugal filter devices fitted with Ultracel membranes (10,000 Da molecular weight cutoff; Merck Millipore). Samples were centrifuged at 2,000 *× g* for 15 min to obtain protein-free ultrafiltrates in the filtrate reservoir. The ultrafiltrate and corresponding total plasma samples were analyzed using the same high-performance liquid chromatography (HPLC)-based analytical method for determination of ceftiofur-related equivalent metabolites. Briefly, 500 μL of the plasma sample was mixed with 7 mL of dithioerythritol solution and incubated in a 50°C water bath for 15 min, with vortex mixing every 5 min. After cooling to room temperature, 1.0 mL of 14% iodoacetamide solution was added, followed by derivatization in the dark for 30 min. The sample pH was then adjusted to 2.5–2.6 with 25% phosphoric acid, and the mixture was centrifuged at 8,000 r/min for 15 min at 4°C. The supernatant was purified using an HLB solid-phase extraction cartridge, eluted with methanol, evaporated under nitrogen at 40°C to below 0.2 mL, and reconstituted in 500 μL of 0.01 mol/L ammonium acetate solution. After filtration through a 0.22-μm nylon membrane, the filtrate was subjected to reverse-phase HPLC analysis on a ZORBAX Stable Bond-C18 column (4.6 mm × 150 mm, 5 μm). The mobile phase consisted of 0.1% trifluoroacetic acid in water and acetonitrile (86:14, vol/vol), with detection at 266 nm, a flow rate of 1.0 mL/min, a column temperature of 30°C, and an injection volume of 50 μL. Concentrations of the unbound fraction were determined from a calibration curve prepared in fortified blank ultrafiltrates.

The unbound fraction (*fu*) was calculated as the ratio of the drug concentration measured in the ultrafiltrate to that measured in total plasma (*fu* = C_ultrafiltrate / C_total). Protein binding (%) was calculated as (1 − *fu*) × 100. The mean *fu* across the three concentrations was used for subsequent PK-PD analyses and Monte Carlo simulations. For urine concentrations, it is assumed that protein binding of ceftiofur and ceftiofur equivalents is negligible (19); therefore, analysis of ceftiofur protein binding in urine was not necessary.

### Pharmacokinetic data

The literature was searched for ceftiofur pharmacokinetic data in dogs (Table 1). Data generated with a slow-release form of ceftiofur used in livestock (ceftiofur crystalline free acid) was excluded from the analysis. There were three studies for each route of administration. Table 1 includes the information on the reported mean values and variances for the study; the dose, the number of subjects, and values for elimination half-life (t ½); maximal (peak) plasma concentration (C_max_); terminal elimination rate constant (λ_z_); total body clearance (CL); and volume of distribution estimated during the terminal elimination phase (VD_AREA_). In addition, as the data from Wang (2011) and Xue (8) were obtained from the China Agricultural University thesis database, the original pharmacokinetic data sets are provided in Table S1 and Table S2 for ease of reference.

**TABLE 1:** Summary of pharmacokinetic parameters of ceftiofur in dogs^*a*^

Source	Number of animals	Dose (mg/kg)	Parameter					Route of administration	Weight (kg)	Sex
			C_max_ (μg/mL)	λ_z_ (h^−1^)	VD_AREA_ (L/kg) or VD_AREA_/F (L/kg)	CL (L/h/kg)	t_1/2_ (h)			
(20)	5	5	19.55 ± 1.18	0.09 ± 0.01	0.43 ± 0.069	0.04 ± 0.004	7.40 ± 0.79	IV	3.4 ± 0.6	Three males and two females
(8)	8	2.2	13.7 ± 0.63	0.07 ± 0.01	0.40 ± 0.075	0.03 ± 0.006	10.24 ± 1.65	IV	6.6–14.4	Four males and four females
Wang, 2011	6	3	20.98 ± 3.75	0.08 ± 0.02	0.24 ± 0.106	0.02 ± 0.01	8.89 ± 2.27	IV	4–8	Three males and three females
LS mean					0.356 ± 0.054	0.029 ± 0.004				
(20)	5	5	10.50 ± 0.22	0.09 ± 0.02	0.47 ± 0.085	0.043 ± 0.09	7.91 ± 1.53	SC	3.4 ± 0.6	Three males and two females
(9, 21)	5	2.2	11.20 ± 8.08	0.17 ± 0.01	0.23 ± 0.06	0.02 ± 0.005	4.12 ± 0.17	SC	10.3–13.5	Five males
(8)	8	2.2	9.03 ± 1.85	0.07 ± 0.01	0.34 ± 0.07	0.025 ± 0.006	9.79 ± 1.55	SC	6.6–14.4	Four males and four females
LS mean					0.345 ± 0.052	0.029 ± 0.019				

^*a*^ C_max_, maximal (peak) plasma concentration; λ_z_, terminal elimination rate constant. VD_AREA_ or VD_AREA_/F, volume of distribution; CL, total body clearance; t ½ , terminal half-life (hr); LS mean is the mean for clearance and volume of distribution calculated to account for within- and between-study variability and the unbalanced number of subjects in each study (reference 19). LS mean values were used in the Monte Carlo simulation.

To obtain an unbiased estimate of averages and variability for the critical parameters of clearance and volume of distribution used in our simulations, in the face of study imbalance, the least squares (LS) mean and its associated variability were calculated (22). To calculate the variability in the LS means, the sums of squares were determined within each study, multiplying the squared standard deviation (Std Dev) of that investigation by its corresponding degrees of freedom. This reflected the within-study sums of squares. Between-study sums of squares likewise reflected the squared difference between the cross-study LS mean value and the mean for the investigation. Within each study, this estimate was multiplied by the number of observations. For the total variability, the within- and between-study variability were added, and the sum was then divided by the overall degrees of freedom (defined as total number of observations, N) across all studies (22). These pharmacokinetic data were used for our simulations described below and are included in Table 1.

### Monte Carlo simulations to determine the pharmacokinetics/pharmacodynamic cutoff (CO_PD_)

Monte Carlo simulations (12, 23) were performed to determine the probability of target attainment (PTA), defined as the probability that unbound drug concentrations achieve the PK-PD target across a range of ceftiofur MIC values for the target pathogen. The PTA was calculated for a 24-h dosing interval based on the administration of an approved label dose of 2.2 mg/kg once daily to dogs. We simulated both the approved SC route of administration, and for comparison, following IV administration since IV administration may occasionally be used in dogs.

For β-lactam antimicrobials, the percentage of time that free (*f*, unbound) drug concentrations exceed the pathogen’s MIC (*f*T >MIC) is the critical PK-PD parameter for predicting the clinical response (24). The LS-mean pharmacokinetic values (described above) were used to generate 10,000 concentration versus time profiles via Monte Carlo simulations (Crystal Ball, Oracle Software, Denver, CO, version 11.1.3.0.0). The values of MIC, T½, VD_AREA_, CL, dose intervals, dose, and *f* were used to estimate *f*T >MIC based upon equation 1,


(1) $$f\%T>MIC=Ln\left(\frac{Dose\times fu}{\left[VD_{AREA}\times MIC\right]}\right)\times \left(\frac{VD_{AREA}}{CL}\right)\times \left(\frac{100}{DI}\right)$$

in which Ln is the natural logarithm, *fu* is the fraction unbound (1 − % protein binding), VD_AREA_ is the apparent volume of distribution, CL is systemic clearance, and DI is the dose interval. Population variability in protein binding was not included in the assessments because such estimates would negatively bias our predictions (25, 26). A target of *f*T >MIC of 50% was used for PTA calculations, consistent with other studies of β-lactam antimicrobial agents (23, 27–30). This value corresponds approximately to a 1-log decline in bacterial counts in the neutropenic mouse thigh infection and other infection models. The susceptible CO_PD_ is determined on the basis of the MIC value for which a 90% PTA is obtained based on the simulated animal population following administration of a specified given dose (12, 23, 30–32).

For our Monte Carlo simulations, we also entered the variability of the mean pharmacokinetic values. Because the calculated variability was small (only 14% coefficient of variation [CV] for some parameters), we used an inflated variance of 30% CV. An inflated variance is commonly used when transferring data from a narrow population of healthy subjects to a clinical population in which the variability is higher (30).

### Establishment of the clinical cutoff (CO_CL_)

Twenty-four Beagle dogs (1–3 years old, 12 males and 12 females; mean body weight 12.5 kg) were acclimated for 1 week prior to the study. All were healthy with no evidence of urinary tract infection based on the physical examination and urine culture. The protocol was approved by the Laboratory Animal Welfare and Animal Experimental Ethics Committee of China Agricultural University. The Classification and Regression Tree (CART) and WindoW analyses in this study were primarily used to help identify the MIC range in which cure rates declined markedly, and the sample size met the minimum requirement for the WindoW method. The parameters used in the WindoW method are provided in Table 2, and the analysis was performed with reference to the methodology described in CLSI VET02 (16). Although the number of animals may have been relatively limited for CART analysis, this was balanced against animal welfare considerations. The final CO_CL_ was, therefore, determined by integrating the results of CART, WindoW, nonlinear regression, and the overall trend in clinical outcomes.

**TABLE 2:** Clinical cure outcomes and statistical indices used for breakpoint determination^*a*^

MIC	Success	Failure	Total	%Success ≤ MIC	%Success > MIC	MaxDiff	AUCSucc	AUCTotal	CAR
0.5	4	0	4	100	56.25	43.75	1	1	1
1	4	0	4	100	41.67	58.33	2.75	3	0.92
2	3	1	4	91.67	25	66.67	5.25	7	0.75
4	2	2	4	81.25	0	81.25	8.25	15	0.55
32	0	4	4	65	0	65	22.25	127	0.18

^*a*^ MIC: minimum inhibitory concentration; %Success ≤ MIC: cumulative cure rate at or below the MIC; %Success > MIC: cumulative cure rate above the MIC; MaxDiff: maximum difference between the two cure rates; AUCSucc: area under the success curve; AUCTotal: area under the total curve; CAR: cumulative area ratio (AUCSucc/AUCTotal).

After sedation, a sterile urinary catheter was placed, and 0.1% salicylic acid in ethanol was instilled into the bladder for 10 min, followed by drainage and saline flushing. After 24 h, 10^8^ colony-forming unit (CFU)/mL of *E. coli* was instilled. On day 7, urine was cultured, and dogs with >10^3^ CFU/mL *E. coli* were considered successfully modeled.

Dogs were randomly assigned to six groups (*n* = 4): one control (saline) and five experimental groups inoculated with isolates of different MICs (0.5, 1, 2, 4, and 32 µg/mL). All five challenge isolates were derived from canine clinical urinary tract infection cases. All received ceftiofur sodium (2.2 mg/kg SC, Veterinary Drug Approval No. 031432019) once daily. On day 7 of treatment, urine cultures and clinical signs were assessed. Dogs with negative cultures and mild/no signs were monitored off therapy for 5 days to confirm cure; those with positive cultures and persistent signs were withdrawn and treated with enrofloxacin.

The clinical sign scoring criteria are shown in Table S3. In this study, cure was defined as a clinical sign score of 0 together with a bacterial count of <10^3^ CFU/mL in urine culture using urine collected by cystocentesis after sedation. Differences in cure rates among MIC groups, as well as between male and female dogs, were compared using Fisher’s exact test, and the overall trend in cure rates across increasing MIC levels was assessed using the Cochran-Armitage trend test. Cure rates were analyzed using Classification and Regression Tree (CART) and WindoW (33) methods to identify preliminary breakpoints, with IC_50_ from nonlinear regression defined as the final breakpoint.

### Activity of ceftiofur in canine urine

Given the concerns about how the activity of antibiotics in urine can be influenced by urinary pH and its constituents (19, 34) and because canine urine pH (35) is typically lower than the conditions used for MIC testing, we investigated whether the activity of cephalosporins is affected by the urine pH range anticipated in treated dogs. We searched the literature to determine if there was evidence that the pH of dog urine affects the activity of cephalosporins and investigated studies to define the usual pH of dog urine.

## RESULTS

### MIC distribution and CO_WT_ determination

We analyzed 880 clinical *E. coli* isolates from canine clinical specimens collected from China, with the majority originating from the urinary tract (521/880, 59.20%), followed by the reproductive system (1/880, 13.18%) and skin (94/880, 10.68%). The ceftiofur MICs for these isolates ranged from ≤0.125 to >16 µg/mL.

The MIC distributions were analyzed with the ECOFFfinder, as described above. For the 519 (out of 880) canine *E. coli* isolates collected from China with definable MIC values (0.125–16 μg/mL), ECOFFinder analysis of the fitted wild-type MIC distribution yielded a 97.5% ECOFF of 1 μg/mL and a 99.0% ECOFF of 2 μg/mL (Table S4). After merging the data from China and the publicly available EUCAST *E. coli*-ceftiofur MIC distributions, these percentile values remained unchanged (Table S4). Analysis of both sets of data was in agreement.

Following EUCAST SOP 10.1 guidance, both the 97.5% and 99% results were reviewed alongside the aggregated histogram to visually confirm that the putative wild-type mode was unimodal and symmetrically bounded. To avoid potential truncation of the wild-type tail, the more conservative estimate (1 μg/mL) was selected as the CO_WT_ for ceftiofur against canine *E. coli* (Fig. S1).

This cutoff provided a method for distinguishing wild-type isolates (MIC ≤1  µg/mL) from those likely to be carrying acquired resistance mechanisms (MIC  ≥2 µg/mL). Based on this cutoff, approximately 93.4% (34,440/36,858) of isolates within the EUCAST data would be classified as wild-type, which is broadly in line with the population proportion typically captured by a 97.5% ECOFF. Therefore, 1 μg/mL was accepted as the ceftiofur *E. coli* CO_WT_ value in all subsequent breakpoint analyses.

### PK-PD breakpoint (CO_PD_) estimation

Plasma protein binding of ceftiofur-related residues was determined in canine plasma prior to PK-PD analysis. Across low (1 µg/mL), medium (8 µg/mL), and high plasma (15 µg/mL) concentrations, the unbound fraction remained consistent, ranging from 16.66% to 18.36%, with a mean *fu* of approximately 17%. This experimentally determined free fraction was applied in all subsequent PK-PD analyses and simulations.

**Fig 1:**
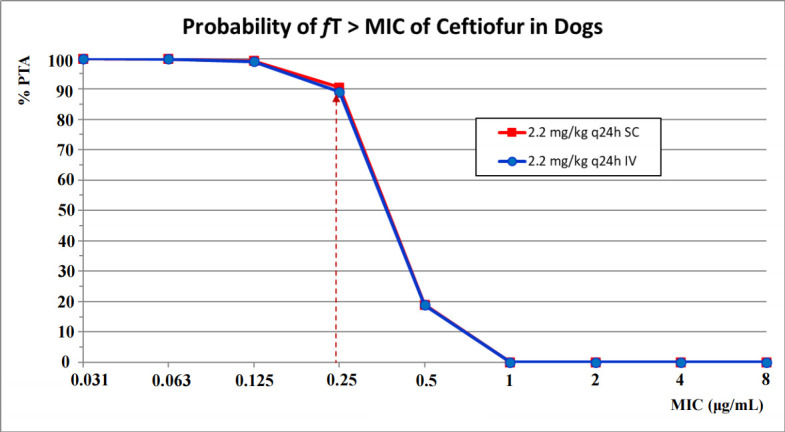
Probability of target attainment (PTA) based on systemic plasma exposure using Monte Carlo simulations (10,000 trials) for ceftiofur administered IV or SC to dogs at a dose of 2.2 mg/kg. The red dashed arrow indicates that the MIC for a PTA of 90% is 0.25 µg/mL, corresponding to 2.2 mg/kg SC once every 24 h.

Our PK-PD analysis and Monte Carlo simulation showed that a 90% PTA could be attained for ceftiofur administered at a dose of 2.2 mg/kg once daily either SC or IV for an MIC value of 0.25 µg/mL (Fig. 1 and Table S6). The two curves are almost superimposable (Fig. 1). Therefore, whether the route is SC or IV, the same breakpoint for susceptibility testing can be used.

An MIC value of ≤0.25 µg/mL produced an *f*T >MIC of 50% or greater. Because this value falls within the wild-type distribution (Fig. S1), we selected a more conservative susceptible value of ≤0.12 µg/mL as our suggested breakpoint, as recommended in the VET02 guidelines (16) and justified in our discussion below.

### Clinical cutoff (CO_CL_) estimation

The observed cure rates were 100%, 100%, 75%, 50%, and 0% for canine bladder infections caused by *E. coli* isolates associated with MIC values of 0.5, 1, 2, 4, and 32 µg/mL, respectively. Although most pairwise comparisons were not significant because of the small group size, cure rates showed a significant decreasing trend with increasing MIC values. For infections caused by isolates with MICs of 0.5 and 1 μg/mL, all dogs reached a clinical score of 0 by day 6 and remained culture-negative on both day 7 and day 12. In the 2 μg/mL group, three of four dogs also had a clinical score of 0 from day 6 onward and remained culture-negative, whereas one dog showed recurrence with positive urine culture on day 12 (Table S5). In three dogs, urine cultures remained positive despite absent clinical signs. Cure rates were lower in males, but not significantly.

**Fig 2:**
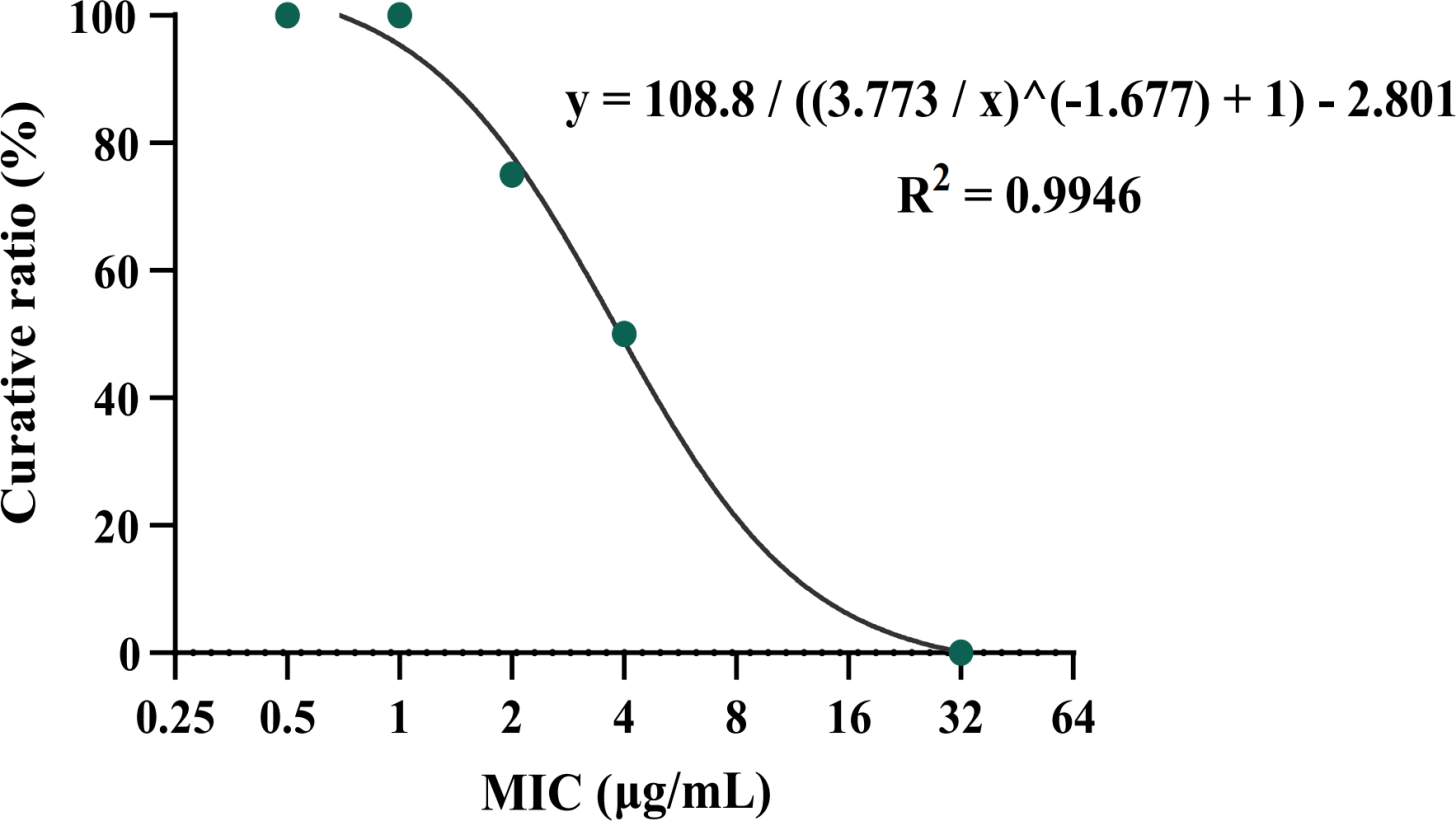
The nonlinear regression analysis of cure rates in relation to the MIC.

For CO_CL_, the cure outcomes across MIC groups were analyzed by CART using Log_2_ MIC and information gain. The tree split at 2.8 µg/mL: MIC <2.8 µg/mL had 91.7% success vs 25% at MIC ≥2.8 µg/mL, indicating 2 µg/mL. In parallel, the WindoW method (33) in Excel identified a CO_CL_ of 4 µg/mL (Table 2). When methods differed, a “breakpoint window” of 2–4 µg/mL was designated. Nonlinear regression of MIC vs cure showed a turning point at 3.8 µg/mL (Fig. 2). Therefore, 2 µg/mL was selected as the final CO_CL_ as the more conservative two-fold dilution within this window, whereas selecting 4 µg/mL would correspond to a relatively low predicted cure rate.

### Activity of ceftiofur in canine urine

Our search for the activity of ceftiofur in canine urine did not reveal any results. However, other papers reported on the activity of various other cephalosporins in urine at varying pH (36, 37). Our investigation found that cephalosporin activity is generally not affected in the pH range of 5–8 (36). Cephalosporins are considered “acid friendly antibiotics,” and one study showed that their activity is improved at low pH (37). We then examined evidence for the typical pH range of canine urine and found that most dogs (male and female) have urine in the pH range of 5.5–8 (35). Therefore, the pH of canine urine will not affect the activity of ceftiofur to change our recommendations for a susceptible MIC value for testing *E. coli* urine isolates for activity against ceftiofur.

### Proposed MIC and disk-diffusion breakpoints for ceftiofur in canine *E. coli*

According to the CLSI VET02 guideline (16), when two cutoff values (e.g., CO_WT_ and CO_PD_ or CO_WT_ and CO_CL_) are available, the suggested breakpoint should be determined following the ranking rule among the available cutoffs. In this study, the CO_WT_ for ceftiofur was 1 µg/mL, the CO_PD_ was 0.25 µg/mL, and the urinary CO_CL_ was 2 µg/mL. Therefore, for the systemic breakpoint, since CO_WT_ >CO_PD_, one dilution lower than the CO_PD_ was selected to avoid a breakpoint falling within the wild-type distribution. This yielded interpretive categories of ≤0.125 µg/mL (S, susceptible), 0.25 µg/mL (I, intermediate), and ≥0.5 µg/mL (R, resistant) following a 2.2 mg/kg SC or IV daily dose. Since drug concentrations in urine are expected to exceed those in plasma, we suggest a breakpoint for canine UTI on the basis of CO_WT_ (i.e., CO_CL_ > CO_WT_). Consequently, the suggested urinary breakpoints are 1/2/4 µg/mL for S/I/R, respectively. Given that our clinical trial was conducted in the context of UTIs, these values are the recommended breakpoints and interpretive criteria for ceftiofur against canine urinary *E. coli* isolates but should not be applied to systemic (non-urine) infections.

**Fig 3:**
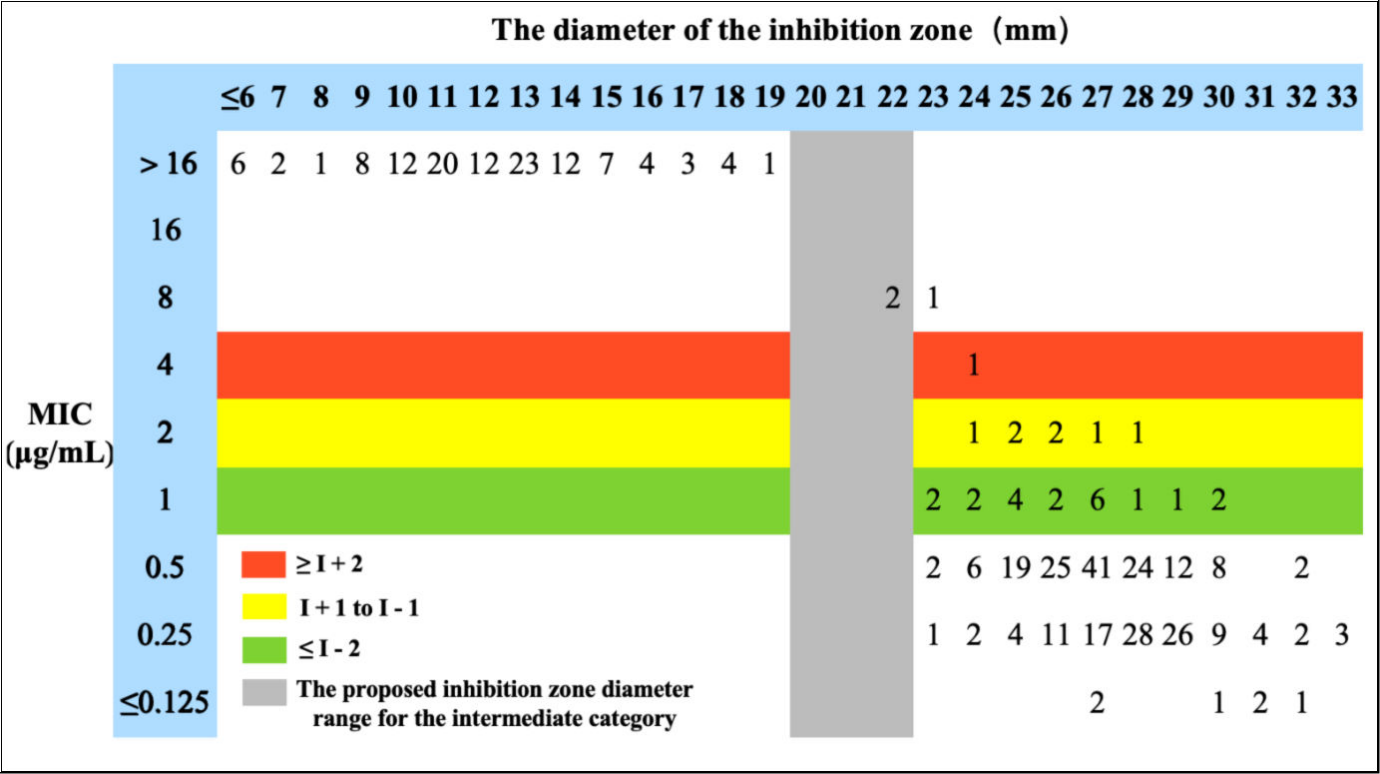
Determination of the optimal inhibition zone diameter using the error rate-bounded (ERB) method. The red area indicates I + 2, the green area indicates I – 2, the yellow area spans I – 1 to I + 1, and the gray area represents the proposed intermediate zone diameter range.

Zone-diameter breakpoints were derived with the error rate-bounded (ERB) method. Wild-type isolates produced ceftiofur inhibition zones ≥ 26.8  mm, whereas non-wild-type isolates measured ≤22.9  mm. Statistical evaluation was performed based on the proposed urinary MIC breakpoints of ≤1 µg/mL (S), 2 µg/mL (I), and ≥4 µg/mL (R). For the 30 μg ceftiofur disk (Fig. 3), the corresponding urinary zone diameter breakpoints were as follows: ≥23 mm (S), 20–22 mm (I), and ≤19 mm (R). All categorical error rates fell within CLSI-acceptable limits (Table S7 and Table S8), confirming that the selected zone diameters satisfy established performance criteria.

Our final proposed breakpoints for ceftiofur administered to dogs are presented in Table 3.

**TABLE 3:** Suggested interpretive categories and breakpoints for testing ceftiofur susceptibility of *E. coli* isolated from dogs^*a*^

	Interpretive categories and MIC breakpoints (µg/mL)			Interpretive category zone diameter breakpoints (nearest whole mm)			Dosage
	S	I	R	S	I	R	
*Escherichia coli*	≤0.125	0.25	≥0.5	–	–	–	2.2 mg/kg SC or IV, every 24 h
*Escherichia coli* (urine)	≤1	2	≥4	≥23	20–22	≤19	2.2 mg/kg SC or IV, every 24 h

^*a*^ The value listed in each cell is the breakpoint (µg/mL) for each interpretive category. S, susceptible; I, intermediate; and R, resistant; –, not applicable. The dose corresponding to the S category is listed. The zone diameter refers to the corresponding zone diameter from an agar disk diffusion test performed according to CLSI standards (CLSI, 2024) and a disk size of 30 µg.

## DISCUSSION

The objective of this investigation was to develop breakpoints for interpreting ceftiofur susceptibility in canine *E. coli* isolates according to CLSI guidelines (16). To our knowledge, this is the first report proposing ceftiofur interpretive criteria specifically for canine *E. coli*. To date, the absence of canine ceftiofur breakpoints in CLSI tables has been a significant gap in veterinary antimicrobial susceptibility testing. Ceftiofur sodium is approved by the U.S. FDA for treatment of canine UTIs at a dose of 2.2 mg/kg administered once daily via SC injection, even though breakpoints were not established at that time. Ceftiofur sodium is also administered in China at a rate of 2.2 mg/kg once daily, but some veterinarians have administered it extralabel for systemic (non-urine) infections without realizing that most isolates will be clinically resistant. The availability of canine-specific breakpoints is particularly important in light of recent reports of resistance to third-generation cephalosporins in canine *E. coli*, with resistance rates to cefovecin ranging from 11.6% to 28.2% in Germany, South Korea, and Poland (38, 39).

The process used to establish these suggested breakpoints aligns with international guidelines for breakpoint development (14). According to the EUCAST SOP 10.1, we evaluated both percentiles and visually reviewed the MIC histogram, ultimately selecting the 97.5^th^ percentile as the most appropriate CO_WT_ value. Based on this approach, the wild-type distribution yielded an ECOFF of 1 µg/mL for *E. coli*, which is consistent with the EUCAST MIC distribution database value for ceftiofur (https://www.eucast.org/). The ECOFF is a microbiological boundary beyond which isolates likely harbor acquired resistance; while not equivalent to breakpoints, ECOFFs are an essential basis for breakpoint setting and surveillance (40).

Establishing CO_PD_ requires multiple data sets, including PD metrics, PK-PD indices, protein binding, and target animal pharmacokinetics (12). In China, both SC and IV formulations of ceftiofur sodium are clinically available and commonly used in veterinary practice. Although both routes are included in our analysis, we acknowledge that SC administration is more commonly used because it is more practical for daily use in the veterinary clinical setting. Our analysis showed that because of rapid absorption of the SC injection and similar pharmacokinetics, both administration routes produced nearly identical CO_PD_ results. Using PK-PD analysis and Monte Carlo simulation, we evaluated the % *f*T >MIC necessary to increase the likelihood of drug efficacy and adopted a conservative threshold of *f*T >MIC of ≥50%, a target that is consistent with that for cephalosporins in the treatment of infections caused by gram-negative bacteria (23, 27–30, 41).

Plasma protein binding, determined *in vitro* using canine plasma, produced a fraction unbound (*fu*) concentration of approximately 0.17, which was incorporated into our PK-PD analysis. Published data in other animal species indicate that ceftiofur-related residues can exhibit higher protein binding in adult cattle (often reported in the ~85%–95% range) (42), whereas in pigs, approximately 70% of ceftiofur-related molecules are reported to be reversibly plasma protein-bound (43). Thus, although some interspecies variation in *fu* exists, the value obtained here remains within the range consistent with the generally high protein binding (approximately 70%–90%) of ceftiofur and its major metabolites reported in the product information from the European Medicines Agency.

When considering the estimated values of CO_WT_ = 1 µg/mL and CO_PD_ = 0.25 µg/mL, we concluded that the ceftiofur susceptibility breakpoint (S) for systemic (non-urine) infections against canine *E. coli* should be ≤0.125 µg/mL (Table 3). Although the CO_PD_ was 0.25 µg/mL, we decided that a more conservative breakpoint of one two-fold dilution lower of 0.125 µg/mL was more appropriate. An S breakpoint of ≤0.25 µg/mL falls within the wild-type distribution (Fig. S1), which is ordinarily discouraged (16, 40). We followed the CLSI guidelines for setting breakpoints (16), which suggest that if the CO_PD_ falls at the lower end (i.e., left side) of the MIC distribution, the conservative approach is to consider the bacterial species as intermediate or resistant. Therefore, our decision was to suggest a breakpoint of ≤0.125 µg/mL. This implies that most, if not all, wild-type *E. coli* isolates from dogs will not test “susceptible” using this breakpoint (Fig. 1 and Fig. S1). Therefore, ceftiofur should be discouraged from use in the treatment of systemic (non-urinary tract) infections in dogs. We considered a dose of ceftiofur sodium higher than the approved dose of 2.2 mg/kg per day in dogs in order to reach a PTA for a higher MIC dilution. However, the target safety information available on the product label (7) indicates that at high doses tested in dogs, thrombocytopenia and anemia were observed.

Because this systemic breakpoint is below the CO_WT_ and ceftiofur is most often used for canine UTIs, we conducted a limited clinical treatment study in a canine UTI model. Brown et al. (9) reported plasma and urine disposition of ceftiofur and its metabolites in dogs following subcutaneous administration. They showed that at a dose of 2.2 mg/kg, urinary total ceftiofur concentrations remained above the CO_WT_ of 1 µg/mL for 24 to 48 h. Nevertheless, since there is no PK-PD target established for UTIs (19, 34) and because cephalosporin activity is generally not affected in the pH range of 5–8 (36, 37), the CO_WT_ was selected as the basis for defining the urinary breakpoint. Thus, our canine UTI-specific breakpoint recommendation (S ≤ 1; I  =  2; R  ≥ 4 µg/mL) integrates both the upper limit of achievable exposure and the observed MIC distribution, striking a balance between clinical efficacy and microbiological stringency. We note that these proposed urine breakpoints differ from the approved cefovecin canine urine breakpoints (S ≤ 2; I = 4; R ≥ 8 µg/mL), even though both agents belong to the third‑generation cephalosporins. This contrast highlights the necessity of species‑ and source‑/site‑specific breakpoint determination, rather than adopting a single uniform breakpoint across hosts, antimicrobial agents, and clinical syndromes.

It is important that laboratories use appropriate and up-to-date breakpoints, as emphasized by Durr et al. (10) and others (28, 44). Using outdated or inconsistent criteria can result in suboptimal treatment choices. In addition, the availability of breakpoints may encourage testing ceftiofur against canine urinary isolates when clinically relevant if interpretation criteria become available.

Based on our findings, ceftiofur may be considered for the treatment of urinary tract infections when supported by antimicrobial susceptibility testing results. In contrast, there is a high probability of clinical resistance among most isolates outside of the urinary tract, potentially leading to suboptimal therapeutic selection. We hope that implementation of these proposed breakpoints by clinical laboratories, monitoring programs, standard-setting organizations, and veterinary practitioners will discourage the use of ceftiofur for the treatment of infections outside the urinary tract.

Implementing these ceftiofur breakpoints may improve antimicrobial stewardship in dogs. Because of the absence of susceptibility testing breakpoints for ceftiofur for dogs (11), laboratories have reported ceftiofur results for canine *E. coli* using breakpoints from other animal species, or they do not report at all.

This study has some limitations. Our pharmacokinetic data were collected from healthy dogs. We have not validated these MIC susceptibility testing breakpoints in animals suffering from clinical infections. Ideally, a multicenter study would validate the reproducibility of AST results. In addition, prospective clinical trials are needed to directly correlate clinical outcomes with MIC breakpoints at the recommended dosing regimen. While our breakpoints should be regarded as “proposed” until clinically validated, they nevertheless represent a considerable improvement over the previous absence of any interpretive criteria. If laboratories and surveillance programs can incorporate these breakpoints into their testing, clinical studies can be planned to confirm the proposed breakpoint validity.

### Conclusion

This study proposes the first evidence-based ceftiofur breakpoints for canine *E. coli*. For systemic infection, the following MIC breakpoints are proposed: ≤0.125 µg/mL (S), 0.25 µg/mL (I), and ≥0.5 µg/mL (R). For UTI, the following MIC and zone diameter breakpoints are proposed: ≤1 µg/mL (S), 2 µg/mL (I), and ≥4 µg/mL (R); disk diffusion zone diameters ≥ 23 mm (S), 20–22 mm (I), and ≤19 mm (R). The criteria used to set these breakpoints reflect drug-pathogen biology and achievable susceptibility testing guidelines (16). The availability of these proposed breakpoints will allow more consistent laboratory reporting, an opportunity to improve therapy decisions, and fewer misclassifications than when referring to human or livestock surrogates. As One-Health stewardship demands species-specific standards, we encourage adoption and prospective outcome studies to validate these proposed breakpoints.

## SUPPLEMENTAL MATERIAL

Supplemental figure and tables jcm.00299-26-s0001.docx Fig. S1 and Tables S1 to S5.

ASM does not own the copyrights to Supplemental Material that may be linked to, or accessed through, an article. The authors have granted ASM a non-exclusive, world-wide license to publish the Supplemental Material files. Please contact the corresponding author directly for reuse.
