# Supplementary material for: Veterinary-specific breakpoints for ceftiofur applicable to canine Escherichia coli
Source: J Clin Microbiol. 2026 Jul 27;64(8):e00299-26. doi: 10.1128/jcm.00299-26 (PMC13471802; doi:10.1128/jcm.00299-26)
Supplement: Supplementary file 1 [file jcm.00299-26-s0001.docx]

**SUPPLEMENTARY FIGURES AND TABLES**

**Supplementary Figure 1**. MIC distributions and nonlinear regression curves for *E. coli* isolates collected from China against ceftiofur as taken from the ECOFFinder program: (A) canine isolates (n = 519) and (B) combined dataset including canine and EUCAST isolates (total n = 37377)

**Supplementary Table 1.** Original pharmacokinetic parameters of ceftiofur sodium following intravenous and subcutaneous administration (2.2 mg/kg) in dogs derived from Xue (2019)

| Parameter | Unit | IV-1 | IV-2 | IV-3 | IV-4 | IV-5 | IV-6 | IV-7 | IV-8 | Mean | SD |
| --- | --- | --- | --- | --- | --- | --- | --- | --- | --- | --- | --- |
| λz | 1/h | 0.09 | 0.06 | 0.06 | 0.08 | 0.08 | 0.06 | 0.06 | 0.06 | 0.07 | 0.01 |
| T_1/2λ_ | h | 7.99 | 11.71 | 10.88 | 8.35 | 8.62 | 11.45 | 12.04 | 10.88 | 10.24 | 1.65 |
| T_max_ | h | 0.25 | 0.50 | 0.25 | 0.50 | 0.50 | 0.25 | 0.25 | 0.25 | 0.34 | 0.13 |
| C_max_ | µg/mL | 13.38 | 13.10 | 14.02 | 14.18 | 13.48 | 13.37 | 13.11 | 14.93 | 13.70 | 0.63 |
| AUC_0-t_ | µg·h/mL | 54.66 | 79.15 | 76.94 | 110.94 | 71.52 | 85.30 | 80.36 | 92.75 | 81.45 | 16.28 |
| AUC_0-∞_ | µg·h/mL | 56.56 | 82.16 | 79.96 | 112.85 | 73.08 | 88.13 | 82.89 | 95.88 | 83.94 | 16.40 |
| V_z_ | mL/kg | 448.50 | 452.50 | 431.91 | 234.77 | 374.31 | 412.43 | 460.85 | 360.33 | 396.95 | 75.07 |
| CL | mL/h/kg | 38.90 | 26.78 | 27.51 | 19.50 | 30.10 | 24.96 | 26.54 | 22.95 | 27.15 | 5.71 |
| MRT_0-t_ | h | 7.07 | 9.58 | 9.45 | 9.73 | 7.81 | 9.21 | 8.70 | 9.43 | 8.87 | 0.96 |
|  |  | SC-1 | SC-2 | SC-3 | SC-4 | SC-5 | SC-6 | SC-7 | SC-8 | Mean | SD |
| λz | 1/h | 0.08 | 0.09 | 0.07 | 0.06 | 0.07 | 0.07 | 0.08 | 0.05 | 0.07 | 0.01 |
| T_1/2λ_ | h | 8.97 | 8.05 | 9.31 | 11.45 | 9.87 | 9.32 | 8.64 | 12.71 | 9.79 | 1.55 |
| T_max_ | h | 1.00 | 1.50 | 1.00 | 1.50 | 1.50 | 1.50 | 1.50 | 1.00 | 1.31 | 0.26 |
| C_max_ | µg/mL | 7.12 | 8.73 | 7.01 | 9.34 | 12.87 | 8.65 | 8.58 | 9.92 | 9.03 | 1.85 |
| AUC_0-t_ | µg·h/mL | 60.08 | 80.29 | 68.24 | 111.60 | 98.28 | 96.34 | 102.79 | 108.97 | 90.82 | 19.11 |
| AUC_0-∞_ | µg·h/mL | 62.12 | 81.80 | 69.73 | 115.91 | 101.54 | 99.21 | 105.07 | 111.63 | 93.37 | 19.82 |
| V_z_ | mL/kg | 458.36 | 312.29 | 423.61 | 313.57 | 308.59 | 298.10 | 261.01 | 361.32 | 342.11 | 67.47 |
| CL | mL/h/kg | 35.42 | 26.90 | 31.55 | 18.98 | 21.67 | 22.17 | 20.94 | 19.71 | 24.67 | 6.02 |
| MRT_0-t_ | h | 9.90 | 10.12 | 9.75 | 12.83 | 10.54 | 11.63 | 11.71 | 13.04 | 11.19 | 1.30 |

λz, terminal elimination rate constant; T_1/2λ_, terminal half-life; T_max_, time to peak concentration; C_max_, maximum concentration; AUC_0-t_, area under the concentration–time curve from time zero to the last measurable concentration; AUC_0-∞_, area under the concentration–time curve extrapolated to infinity; V_z_, apparent volume of distribution during the terminal phase; CL, total body clearance; MRT_0-t_, mean residence time.

**Supplementary Table 2.** Original pharmacokinetic parameters of ceftiofur sodium following intravenous administration (2.2 mg/kg) in dogs derived from Wang (2011)

| Parameter | Unit | Dog1 | Dog2 | Dog3 | Dog4 | Dog5 | Dog6 | Mean±SD |
| --- | --- | --- | --- | --- | --- | --- | --- | --- |
| A | µg/mL | 12.22 | 8.02 | 8.83 | 9.49 | 18.46 | 15.23 | 12.04±4.11 |
| α | 1/h | 0.13 | 0.19 | 0.14 | 0.22 | 0.21 | 0.28 | 0.20±0.06 |
| B | µg/mL | 3.63 | 8.51 | 8.48 | 7.96 | 2.38 | 1.90 | 5.48±3.17 |
| β | 1/h | 0.12 | 0.073 | 0.077 | 0.074 | 0.092 | 0.056 | 0.08±0.02 |
| V_c_ | L/kg | 0.19 | 0.18 | 0.17 | 0.17 | 0.14 | 0.17 | 0.17±0.02 |
| t_1/2α_ | h | 5.3 | 3.59 | 5.01 | 3.14 | 3.23 | 2.51 | 3.80±1.11 |
| t_1/2β_ | h | 5.56 | 9.42 | 9.04 | 9.37 | 7.57 | 12.40 | 8.89±2.27 |
| K_21_ | 1/h | 0.13 | 0.14 | 0.10 | 0.14 | 0.11 | 0.08 | 0.12±0.02 |
| K_10_ | 1/h | 0.13 | 0.11 | 0.099 | 0.12 | 0.18 | 0.19 | 0.14±0.04 |
| K_12_ | 1/h | 0.00005 | 0.026 | 0.0089 | 0.038 | 0.014 | 0.060 | 0.02±0.02 |
| AUC | (µg/mL)·h | 122.62 | 157.11 | 174.59 | 150.65 | 112.05 | 89.20 | 134.37±31.84 |
| CLs | 1/kg/h | 0.024 | 0.019 | 0.017 | 0.020 | 0.027 | 0.034 | 0.02±0.01 |

A and B, intercepts of the distribution and elimination phases, respectively; α and β, hybrid first-order rate constants of the distribution and elimination phases (h^-1^); t_1/2α_ and t_1/2β_, half-lives of the distribution and elimination phases, respectively; V_c_, apparent volume of the central compartment; k_12_, transfer rate constant from the central to the peripheral compartment; k_21_, transfer rate constant from the peripheral to the central compartment; k_10_, elimination rate constant from the central compartment; AUC, area under the concentration–time curve; CLs, systemic clearance.

**Supplementary Table 3.** Criteria for scoring urinary clinical signs in dogs.

| Symptoms | 0 points (none) | 1 point (mild) | 2 points (moderate) | 3 points (severe) |
| --- | --- | --- | --- | --- |
| Pollakiuria | Normal urination frequency | Slightly increased urination frequency, with a mild increase in the number of voids that does not affect daily activity | Markedly increased urination frequency, with occasional urgency | Very frequent urination (hourly or more often), unable to hold urine |
| Stranguria | Smooth urination | Slightly prolonged urination time or slightly prolonged urination posture | Obvious straining during urination, requiring repeated attempts or inability to empty completely | Repeated attempts with difficulty passing urine, extremely small urine volume, persistent straining with almost no urine output |
| Dysuria | No signs of pain | Mild discomfort during urination, such as hesitation or pausing | Obvious discomfort, interrupted urination, whining, or hesitancy | Severe pain, crying out during urination or refusing to urinate |
| Hematuria | Clear urine with no visible blood | Occasional pink discoloration; trace blood | Obvious hematuria, frequent blood in urine | Marked hematuria, dark red urine, often with blood clots or blood fragments |
| Incontinence | No leakage; full control | Occasional minimal urine leakage (eg, dribbling during sleep) | Frequent small-volume leakage, with wet spots or mild soiling in daily life | Unable to control urination, with nearly continuous leakage |
| Urine odor | No abnormal odor | Slightly stronger urine odor | Obvious abnormal odor, noticeable change in smell | Strong pungent abnormal odor, persistent without improvement |

**Supplementary Table 4**. ECOFFinder Output Summary for CO_WT_ Determination for MIC data collected from China and the combined dataset including EUCAST data.

|  | Selected Values | Exact | R'd-up | %Obs> | %ECOFF |
| --- | --- | --- | --- | --- | --- |
| Canine isolates  (n = 519) | ECOFF 95.0% | 0.7818 | 1 | 6.0% | 13.6% |
|  | ECOFF 97.5% | 0.9726 | 1 | 6.0% | 13.6% |
|  | ECOFF 99.0% | 1.2538 | 2 | 4.6% | 2.1% |
|  | ECOFF 99.5% | 1.4905 | 2 | 4.6% | 2.1% |
|  | ECOFF 99.9% | 2.1291 | 4 | 3.7% | 0.1% |
| Combined dataset (EUCAST data and canine isolates)  (n = 37377) | ECOFF 95.0% | 0.7818 | 1 | 6.6% | 13.6% |
|  | ECOFF 97.5% | 0.9726 | 1 | 6.6% | 13.6% |
|  | ECOFF 99.0% | 1.2538 | 2 | 6.1% | 2.1% |
|  | ECOFF 99.5% | 1.4905 | 2 | 6.1% | 2.1% |
|  | ECOFF 99.9% | 2.1291 | 4 | 4.9% | 0.1% |

ECOFFs were estimated using non-linear regression in the ECOFFinder tool. Rounded-up (R'd-up) values are used to propose practical clinical ECOFFs. %Obs> indicates the proportion of isolates with MICs above the proposed cutoff; %ECOFF reflects the non-wild-type rate under each candidate ECOFF threshold.

**Supplementary Table 5.** Daily clinical scores and urine culture results of dogs in different MIC groups during treatment and follow-up.

| No. | Sex | Group | Day 1 Score | Day 2 Score | Day 3 Score | Day 4 Score | Day 5 Score | Day 6 Score | Day 7 Score | Day 7 Culture | Day 8 Score | Day 9 Score | Day 10 Score | Day 11 Score | Day 12 Score | Day 12 Culture |
| --- | --- | --- | --- | --- | --- | --- | --- | --- | --- | --- | --- | --- | --- | --- | --- | --- |
| 1 | Male | MIC 0.5 | 8 | 6 | 5 | 4 | 2 | 0 | 0 | Negative | 0 | 0 | 0 | 0 | 0 | Negative |
| 2 | Male | MIC 0.5 | 11 | 7 | 6 | 3 | 0 | 0 | 0 | Negative | 0 | 0 | 0 | 0 | 0 | Negative |
| 3 | Female | MIC 0.5 | 8 | 6 | 3 | 1 | 0 | 0 | 0 | Negative | 0 | 0 | 0 | 0 | 0 | Negative |
| 4 | Female | MIC 0.5 | 3 | 1 | 0 | 0 | 0 | 0 | 0 | Negative | 0 | 0 | 0 | 0 | 0 | Negative |
| 5 | Male | MIC 1 | 11 | 8 | 6 | 4 | 2 | 0 | 0 | Negative | 0 | 0 | 0 | 0 | 0 | Negative |
| 6 | Male | MIC 1 | 4 | 4 | 0 | 0 | 0 | 0 | 0 | Negative | 0 | 0 | 0 | 0 | 0 | Negative |
| 7 | Female | MIC 1 | 5 | 5 | 2 | 1 | 0 | 0 | 0 | Negative | 0 | 0 | 0 | 0 | 0 | Negative |
| 8 | Female | MIC 1 | 7 | 5 | 3 | 0 | 0 | 0 | 0 | Negative | 0 | 0 | 0 | 0 | 0 | Negative |
| 9 | Male | MIC 2 | 2 | 1 | 1 | 0 | 0 | 0 | 0 | Negative | 0 | 0 | 0 | 0 | 0 | Negative |
| 10 | Male | MIC 2 | 4 | 4 | 3 | 3 | 1 | 0 | 0 | Negative | 0 | 0 | 0 | 1 | 1 | Positive |
| 11 | Female | MIC 2 | 4 | 4 | 3 | 1 | 0 | 0 | 0 | Negative | 0 | 0 | 0 | 0 | 0 | Negative |
| 12 | Female | MIC 2 | 7 | 7 | 5 | 3 | 0 | 0 | 0 | Negative | 0 | 0 | 0 | 0 | 0 | Negative |
| 13 | Male | MIC 4 | 8 | 8 | 5 | 2 | 2 | 1 | 0 | Positive | NA | NA | NA | NA | NA | NA |
| 14 | Male | MIC 4 | 4 | 4 | 2 | 1 | 1 | 0 | 0 | Negative | 0 | 0 | 0 | 0 | 0 | Positive |
| 15 | Female | MIC 4 | 4 | 4 | 4 | 3 | 3 | 1 | 0 | Negative | 0 | 0 | 0 | 0 | 0 | Negative |
| 16 | Female | MIC 4 | 1 | 1 | 0 | 0 | 0 | 0 | 0 | Negative | 0 | 0 | 0 | 0 | 0 | Negative |
| 17 | Male | MIC 32 | 6 | 5 | 5 | 2 | 2 | 2 | 1 | Positive | NA | NA | NA | NA | NA | NA |
| 18 | Male | MIC 32 | 7 | 6 | 6 | 3 | 3 | 2 | 2 | Positive | NA | NA | NA | NA | NA | NA |
| 19 | Female | MIC 32 | 3 | 2 | 1 | 1 | 0 | 0 | 0 | Negative | 0 | 0 | 0 | 0 | 0 | Positive |
| 20 | Female | MIC 32 | 5 | 5 | 4 | 3 | 2 | 1 | 1 | Positive | NA | NA | NA | NA | NA | NA |
| 21 | Male | Control | 1 | 1 | 0 | 0 | 0 | 0 | 0 | Negative | 0 | 0 | 0 | 0 | 0 | Negative |
| 22 | Male | Control | 0 | 0 | 0 | 0 | 0 | 0 | 0 | Negative | 0 | 0 | 0 | 0 | 0 | Negative |
| 23 | Female | Control | 1 | 0 | 0 | 0 | 0 | 0 | 0 | Negative | 0 | 0 | 0 | 0 | 0 | Negative |
| 24 | Female | Control | 0 | 0 | 0 | 0 | 0 | 0 | 0 | Negative | 0 | 0 | 0 | 0 | 0 | Negative |

NA, not applicable because the dog was withdrawn from follow-up after a positive culture and persistent clinical signs.

**Supplementary Table 6.** Probability of Target Attainment (PTA) for a ceftiofur dose of 2.2 mg/kg administered to dogs IV or SC.

|  |  | **MIC (µg/mL) for Ceftiofur in Dogs** | | | | | | | | | |
| --- | --- | --- | --- | --- | --- | --- | --- | --- | --- | --- | --- |
| **Dosage and Route** | **0.03** | **0.06** | **0.12** | **0.25** | **0.5** | **1** | **2** | **4** | **8** | **16** | **32** |
| 2.2 mg/kg q24h SC | 100 | 99.87 | 99.24 | 90.5 | 18.92 | 0.01 | 0 | 0 | 0 | 0 | 0 |
| 2.2 mg/kg q24h IV | 99.96 | 99.81 | 99.03 | 88.98 | 18.85 | 0 | 0 | 0 | 0 | 0 | 0 |

The top row is the MIC range (µg/mL) and each cell represents the percent PTA. We used a threshold of ≥ 90% PTA for the determination of the PK-PD cutoff (CO_PD_).

**Supplementary Table 7**. Discrepancy Rates Observed in the Present Study

| MIC Range | Total | Very Major | Major | Minor |
| --- | --- | --- | --- | --- |
| ≥I+2 | 118 | 1 (0.85%) | N/A | 2 (1.69%) |
| I+1 to I-1 | 28 | 1 (3.57%) | 0 (0.00%) | 7 (25.00%) |
| ≤I-2 | 252 | N/A | 0 (0.00%) | 0 (0.00%) |
| Total | 398 | 2 (0.50%) | 0 (0.00%) | 9 (2.26%) |

**Supplementary Table 8**. Guidance for Acceptable Discrepancy Rates (With Intermediate Ranges)

| MIC Range | Discrepancy Rates | | |
| --- | --- | --- | --- |
|  | Very Major | Major | Minor |
| ≥I+2 | ＜2% | N/A | ＜5% |
| I+1 to I-1 | ＜10% | ＜10% | ＜40% |
| ≤I-2 | N/A | ＜2% | ＜5% |
